# Cucurbitacin B from Cucurbitaceae Plants: Treating Pancreatic Cancer via Inducing Mitophagy, Inhibiting Glycolysis, and Enhancing Immune Function

**DOI:** 10.3390/nu17172809

**Published:** 2025-08-29

**Authors:** Dongge Yin, Hongyue Chen, Xiaohong Jing, Shuting Lin, Yufei Sun, Rongrong Chang, Yang Feng, Xiaoxv Dong, Changhai Qu, Jian Ni, Xingbin Yin

**Affiliations:** School of Chinese Materia Medica, Beijing University of Chinese Medicine, Beijing 102488, China; 20230941515@bucm.edu.cn (D.Y.); 20240935229@bucm.edu.cn (H.C.); 20230935212@bucm.edu.cn (X.J.); 20240935230@bucm.edu.cn (S.L.); 20230935210@bucm.edu (Y.S.); 20230935211@bucm.edu.cn (R.C.); 20240935283@bucm.edu.cn (Y.F.); dxiaoxv@163.com (X.D.); quchanghai@bucm.edu.cn (C.Q.); njtcm@263.net (J.N.)

**Keywords:** Cucurbitacin B, pancreatic cancer, mitophagy, glycolysis, immunity

## Abstract

Background: Cucurbitacin B (CuB) is a relatively unique and valuable component in plants of the Cucurbitaceae family due to its diverse and remarkable physiological activities, but its specific mechanisms in regulating tumor metabolism and immune response remain unclear. The hypoxic tumor microenvironment (TME) of pancreatic cancer induces metabolic reprogramming in cancer cells, causing them to rely on glycolysis for energy. LDHA, a key enzyme in glycolysis, can suppress glycolysis and tumor growth when inhibited. Objective: The objective of this study was to investigate the mechanism of CuB against pancreatic cancer and its effect on the immune system. Methods: In this study, cell migration/invasion assays, immunofluorescence, ELISA, Western blot, CETSA, flow cytometry, mouse models, and metabolomic and transcriptomic analyses were utilized to systematically elucidate the mechanism by which CuB inhibits pancreatic cancer and activates the immune system. Results: This study confirms that CuB inhibits pancreatic cancer by suppressing the PI3K/Akt/mTOR pathway and activating PINK1/Parkin to induce mitophagy, thereby inhibiting cell migration, invasion, and proliferation. It downregulates the expression of LDHA to block glycolysis, reduce lactate production and efflux, and improve the acidic TME. CuB also induces ICD to activate dendritic cells, promote CD8+ T-cell and M1 macrophage infiltration, and reduce the levels of regulatory T cells. Metabolomic and transcriptomic analyses validate CuB’s dual effects on metabolic reprogramming and immune activation. Conclusions: This study, for the first time, reveals that CuB induces mitophagy via the PI3K/Akt/mTOR and PINK1/Parkin pathways to selectively eliminate damaged mitochondria and suppress tumor energy metabolism. CuB inhibits pancreatic cancer through a triple mechanism—inducing mitophagy, inhibiting glycolysis, and activating immunity—which provides innovative insights for pancreatic cancer therapy.

## 1. Introduction

Cucurbitacin B (CuB) is a unique and physiologically active component found in cucurbitaceous plants, primarily present in their fruits, seeds, or vines. CuB has demonstrated multifaceted effects, including antitumor, anti-inflammatory, antibacterial, and antiviral activities. Additionally, it exhibits antifeedant and repellent properties against certain insects, making it a natural potential plant protectant for crop pest control. Due to its remarkable therapeutic efficacy against various cancers [1,2], several drugs with CuB as the main ingredient or lead compound are currently in clinical trials or have already been applied in clinical treatments.

In the 2022 global cancer statistical analysis, pancreatic cancer ranked as one of the most lethal malignancies worldwide, accounting for 4.8% of all cancer fatalities [3]. Pancreatic ductal adenocarcinoma (PDAC), accounting for 95% of pancreatic cancers, is one of the most invasive malignant tumors [4]. PDAC has numerous fibrogenic mechanism-related cells but poor vascularization, leading to continuous intratumoral hypoxia. Therefore, to ensure energy supply, PDAC cells change from oxidative phosphorylation (OXPHOS) to glycolysis. As a result, most pyruvic acid (PA) is converted to lactic acid (LA) instead of entering the tricarboxylic acid (TCA) cycle [5]. Such glycolytic reprogramming, classically known as the Warburg effect, is pathognomonic of cancer metabolism and prominently observed in PDAC cells [6]. On the one hand, lactate inhibits the functions and viability of T cells and NK cells, aiding immune evasion [7,8]. On the other hand, the enhanced energy metabolism hinders the growth of immune cells by competing with T cells for energy and nutrients within the tumor microenvironment (TME) [9,10]. Lactate dehydrogenase A (LDHA), a key enzyme in aerobic glycolysis, catalyzes the conversion of PA to LA. Thus, it serves as a viable target for tumor therapy, primarily by inhibiting tumor metabolic reprogramming, decreasing lactate secretion, and consequently ameliorating the TME [11]. The special tumor immune microenvironment (TIME) of “cold” tumors severely impairs the efficacy of immunotherapy, characterized by T-cell deficiency or exclusion, accompanied by abundant tumor-promoting macrophages, anti-inflammatory cytokines, and immunosuppressive cells [12,13]. Pancreatic cancer is a typical “cold” tumor, with scarce adaptive immune cells but a significant enrichment of innate immune cells. Therefore, improving the tumor-suppressive immune microenvironment and activating antitumor immune cells are also key to the treatment of pancreatic cancer.

Studies indicate that the antitumor activity of CuB is linked to autophagy [14,15], as it induces DNA damage by elevating ROS levels and activating the PI3K/Akt/mTOR pathway [16]. Research has revealed that the PI3K/Akt/mTOR pathway facilitates the transition to aerobic glycolysis and reprogramming of glucose metabolism [17]. By inhibiting mTORC1, the expression of LDHA and other metabolic effectors can be downregulated, and when this is combined with a PI3K inhibitor, the inhibitory effect on cancer cells is enhanced [18]. Furthermore, CuB has been shown to modulate immune responses, specifically by reducing the expression of cytokines such as NOS, COX-2, and MHC-II in macrophages [19]. Therefore, given these pleiotropic effects and the urgent need for novel therapeutics in pancreatic cancer, CuB is an intriguing candidate for targeting both tumor metabolism and immune dysregulation. However, the exact mechanisms by which CuB modulates tumor metabolism and immune responses in pancreatic cancer are not well understood.

Here, we report the CuB-induced mitophagy of pancreatic cancer via the PI3K/Akt/mTOR and PINK1/Parkin pathways, subsequently modulating the key enzyme levels in the glycolysis pathway and reducing the energy supply for pancreatic cancer cells. In particular, the downregulation of LDHA expression decreases the conversion of PA to LA, and the TME is also improved, with an increase in the infiltration of immune cells. Therefore, we reveal that CuB can induce cellular mitophagy, inhibit glycolysis in pancreatic cancer cells, and improve the TME in terms of metabolic balance through the PI3K/Akt/mTOR and PINK1/Parkin pathways and LDHA downregulation. Significantly, LDHA serves as a promising target for CuB to exert its effects in inhibiting glycolysis and enhancing the TME.

## 2. Materials and Methods

### 2.1. Cell Culture

Human pancreatic cancer cells (PANC-1) (MeilunBio, Dalian, China) and mouse PANC-02 cells (Procell, Wuhan, China) were cultured in Dulbecco’s Modified Eagle Medium (DMEM; Gibco, NY, USA, C11995500BT) supplemented with 10% fetal bovine serum (FBS; Corning, MA, USA, 35-081-CV) and 1% penicillin–streptomycin–amphotericin B (Biorigin, Beijing, China, BN28053) and incubated in an incubator set at 37 °C with a carbon dioxide concentration of 5%.

### 2.2. Cell Viability Determination

PANC-1 cells were seeded in 96-well plates and treated with DMEM or CuB for 24, 48, or 72 h. The optical density at 450 nm was measured following the guidelines of Cell Counting Kit-8 (cck-8 kit) (NCM Biotech, Suzhou, China, C6005).

In the colony formation experiment, cells were seeded in 6-well plates and treated with DMEM or CuB (Shanghai YuanYe Bio-Technology Co., Ltd., Shanghai, China, ≥98%, LOT: J16IB220291). After 24 h, cells in different groups were transferred into new 6-well plates (1000 cells/well). After being washed twice with cold PBS, the colonies were stored in fixative and stained with 0.1% crystal violet. Then, these colonies were counted, and images were taken of them.

In the wound healing experiment, a 96-well plate was used. A scratch was created in each well when its cell density was about 90%. Before applying the DMEM or CuB treatment at different concentrations, we washed the cells twice with 1 × PBS to reduce the impact of cell proliferation on the results. The wound healing process in the same area was observed using an inverted light microscope at 0 and 24 h.

The invasion ability of PANC-1 cells was assessed using a Matrigel^®^ Invasion Chamber (Corning, Inc., Lowell, MA, USA). The upper chamber was filled with 200 µL of DMEM or varying CuB concentrations, while the lower chamber contained 500 µL of medium supplemented with 10% FBS. Following a 24-h incubation period, cells from both chambers were fixed with 4% paraformaldehyde for 15 min. After two washes with PBS, staining was performed using 0.1% crystal violet. Excess dye was removed by means of repeated PBS washes until the background became clear. Cells remaining in the upper chamber were gently wiped away with a cotton swab. Images were captured using an inverted fluorescence microscope.

### 2.3. DAPI, Calcein AM/PI, and Monodansyl Cadaverine (MDC) Staining

For cellular experiments, adherent cultures in 6-well plates were maintained in DMEM supplemented with various CuB concentrations for 24 h. Following fixation (4% paraformaldehyde, 10 min), nuclei were counterstained with DAPI (Biorigin, Beijing, China, BN20923) and visualized using a Nikon Eclipse Ti Inverted Microscope (Nikon, Japan) after PBS washes. Parallel samples were processed for the viability assessment using the Calcein AM/PI dual-staining kit per the manufacturer’s protocol (Lablead, Beijing, China, C30002), with live/dead cell quantification performed on three independent fields per well.

To evaluate autophagy, an MDC stain kit (Beyotime, Shanghai, China, C3018S) was used to detect acidic vesicle organelles such as autophagosomes. After being treated using the same method as above, cells were stained with MDC for 40 min in the dark at 37 °C. Finally, the cells were washed with a 1 × Buffer and examined using Confocal Laser Scanning Microscopy (CLSM) (Leica SP8, Wetzlar, Germany). The cells were cultured in a 96-well plate using the same treatment method as above, and their fluorescence (Eex/Eem = 335 nm/512 nm) was detected using a fluorescence microplate reader.

### 2.4. Flow Cytometry

Cells were processed using the same method as above and collected. Cell apoptosis rates were quantified by means of flow cytometry using an Annexin V-FITC detection kit (Beyotime, Shanghai, China, C1062L).

### 2.5. Measurement of Cellular ATP Content

ATP levels were detected using the ATP Content Assay Kit (SolarBio, Beijing, China, BC0305). PANC-1 cells were processed using the same method as above. Then, the cell lysate or supernatant was collected. The working reagent was mixed with the cell lysate or supernatant and Reagent 1. Absorption was immediately (within 10 s) measured at 340 nm. The mixed solution was incubated at 37 °C for 3 min before retesting. The ATP content was calculated according to the instruction manual.

### 2.6. Calreticulin (CRT) and High Mobility Group Box 1 (HMGB1) Measurement

After being treated using the same method as above, cells were fixed and then permeabilized with 0.5% Triton. The cells were blocked with 5% BSA, then incubated with anti-HMGB1 (A19529, 1:500) or anti-CRT (ab92516, 1:800) overnight at 4 °C. After washing with cold 1 × PBS, fluorescent secondary antibody was added to the culture plate, and DAPI staining was performed simultaneously. Finally, the fluorescence was observed via CLSM.

The PANC-1 cells were plated on the indicated day of treatment. Then, the cell supernatant or cell lysate was obtained, and its content was measured according to the instructions of the CRT (FineTest, Wuhan, China, EH1659) or HMGB1 (FineTest, Wuhan, China, EH0884) ELISA kit.

### 2.7. Glucose Consumption Assay

After processing using the same method as above, a Glucose Content Assay Kit (SolarBio, Beijing, China, BC2505) was used to treat the cells. The cell sediment was resuspended in distilled water and boiled for 10 min. The supernatant was collected by means of centrifugation and incubated with the working reagent for 15 min. The absorbance was detected using a fluorescence microplate reader at 505 nm.

### 2.8. Lactate Secretion Assay

Cells or supernatants were processed and collected as described above. After ultrasonic lysis, they were treated according to the instructions of the CheKine^TM^ Micro Lactate Assay Kit (Abbkine, Wuhan, China, KTB1100). Their absorbance was detected using a fluorescence microplate reader at 450 nm.

### 2.9. PA Secretion Assay

A pyruvic acid production assay was performed by using the Pyruvate Content Assay Kit (SolarBio, Beijing, China, BC2200). Cells or supernatants were processed and collected as described above and lysed by means of ultrasonication. After the cells were mixed with Reagents 1 and 2 in sequence, their absorbance was measured at 520 nm.

### 2.10. Enzyme Activity Related to Glycolysis

PANC-1 cells were processed and collected as described above. Then, the enzymatic activities of LDHA (FineTest, Wuhan, China, EH2155), PC (Elabscience, Wuhan, China, E-BC-K608-M), and G6PD (FineTest, Wuhan, China, EH1022) in the supernatant were detected according to the instructions of the corresponding reagent kits.

### 2.11. NAD^+^/NADH Ratio Assay

PANC-1 cells were inoculated into a 96-well plate containing 50 μL of culture medium after different treatments according to the instructions of the NAD/NADH-Glo^TM^ Assay (Promega, Madison, MI, USA, G9071). Subsequently, each well was treated according to the kit’s instructions, and the signals were measured using a luminometer.

### 2.12. Extracellular Acidification Rate (ECAR) Detection

PANC-1 cells were processed and collected as described above. Following the instructions for the Extracellular Acidification Rate (ECAR) Fluorometric Assay Kit (Elabscience, Wuhan, China, E-BC-F069), the supernatants were resuspended in distilled water. The cells were seeded in a new 96-well plate and incubated in the dark at 37 °C for 30 min. Immediately after the working solution was added, the fluorescence value was detected with a fluorescence microplate reader (Ex/Em = 490 nm/535 nm). After continued incubation for another 30 min, the fluorescence value was detected once more.

### 2.13. Extracellular Oxygen Consumption Rate (ORC) Detection

The cellular oxygen consumption rate was measured using the Extracellular OCR Plate Assay Kit (Dojindo, Kumamoto, Japan, E297). The cells were seeded in a 96-well plate with a black transparent background. After 12 h, the culture medium or working solution was added and incubated at 37 °C for 30 min in a microplate reader. Mineral oil was immediately added and incubated for 5 min after the drug or culture medium was added. The fluorescence intensity was detected every 10 min for 200 min in continuous reading mode using a microplate reader.

### 2.14. RT-qPCR

RNA extraction was carried out with the SteadyPure Universal RNA Extraction Kit (Accurate, Changsha, China, AG21017). Complementary DNA was created using an Evo M-MLV reverse transcription kit (Accurate, Changsha, China, AG11706). Quantitative PCR analysis was conducted on an iQ5 Real-Time PCR system (Applied Biosystems, Foster City, CA, USA) employing SYBR Green Pro Taq HS Premixed qPCR Kit III (Accurate, Changsha, China, AG11739). All gene expression data were normalized to those for GAPDH, with the primer sequences provided in Supplementary Table S1. All the reagent kits were purchased from Accurate.

LDHA mRNA (transcript number NM_001165414.2) was used as an overexpression vector. The eukaryotic transient vector pRP [Exp]-Neo CMV > hLDHA [NM_001165414.2]: IRES: EGFP (Vector ID: DS10038) and pRP [Exp]-NeoR CMV > EGFP control vector (Vector ID: MJ-282) were constructed by Beijing MIJIA Scientific Co., Ltd. (Beijing, China). After Sanger sequencing, the endotoxin-free plasmid was extracted and prepared for transfection into host cells.

The Lipofect5000 transfection reagent (Lablead, Beijing, China, T2123) was used to transfect LDHA overexpression plasmids into cells. First, according to the instructions from the reagent supplier, the cells were cultured in a complete medium without antibiotics until the cell density reached 80%. The Lipofect5000 and plasmid mixture prepared in fresh medium were then replaced in the wells and cultured for another 36 h.

### 2.15. Cellular Thermal Shift Assay (CETSA)

Logarithmically growing PANC-1 cells were passaged into T75 flasks. When the cell confluency reached 90%, the cells were divided into two groups, treated with either 0.1% (*v*/*v*) DMSO or 50 μg/mL CuB, respectively. After incubation for 60 min following the administration of the treatments, the cells were collected by means of centrifugation and washed with PBS (0.01 M, pH 7.4). To the cell pellet, 1 mL of pre-chilled PBS (0.01 M, pH 7.4) containing 1% PMSF (Solarbio, Beijing, China, P0100) was added, and the mixture was left to stand on ice for 30 min. The cells were distributed into 6 centrifuge tubes (approximately 150 μL per tube) and heated in a metal bath at 42, 47, 52, 57, 62, or 67 °C for 3 min, followed by equilibration at room temperature (RT) for 3 min. Cells were lysed using a repeated freeze–thaw method (vortex for 30 s → freeze in liquid nitrogen for 1 min → thaw in a 37 °C water bath for 3 min → vortex for 30 s; repeated 3 times to ensure complete lysis). After centrifugation at 12,000× *g* at 4 °C for 20 min, the supernatant was transferred to a new EP tube. The supernatant was mixed with 30 μL of loading buffer (Servicebio, Wuhan, China, G3011), vortexed thoroughly, and heated at 99 °C for 5 min for protein denaturation. Finally, the samples were subsequently analyzed via Western blotting (WB).

### 2.16. Animals and Tumor Models

Male C57BL/6N mice (20–22 g) were purchased from Beijing SiPeiFu Laboratory Animal Technology Co. (Beijing, China) The experiment was performed under the guideline of the Laboratory Animal Ethics Committee of Beijing University of Traditional Chinese Medicine (BUCM-2024050702-2069). To establish a mouse model of pancreatic cancer, a 100 μL cell suspension (5 × 10^6^ cells) was subcutaneously injected into the right upper limb of each mouse. When the tumor size reached about 100 mm^3^, the mice were randomly divided into three groups, with 6 mice in each group. The CuB groups were injected with CuB at a concentration of 0.5 mg/mL in 1% DMSO diluted with physiological saline (0.9% NaCl), while the control group was injected with physiological saline (0.9% NaCl) and the DMSO group received 0.1% DMSO as vehicles. Throughout the entire process, the size of the tumors was measured using a vernier caliper, and the tumor volume was calculated using the following formula: volume = 0.5 × L × W × W, where L and W represent the tumor length and width. We also performed HE staining on major organs (heart, liver, spleen, and kidneys) to determine in vivo biosafety; the levels of liver (ALT, AST, and ALP) and kidney (GFR, UREA, and CREA) function indicators were also evaluated.

### 2.17. Immunofluorescence (IF)

Fixed tumor tissues (5 μm sections) were blocked with 5% BSA, then co-incubated with antibodies and DAPI. TUNEL (Servicebio, Wuhan, China, G1504) and ROS (Beyotime, Shanghai, China, S0033S) staining was performed. Antifluorescence quenching mounting medium (Lablead, Beijing, China, D0120) was used. Sections were observed and imaged via CLSM; the fluorescence intensity was quantified using ImageJ (ImageJ 1.54f). Image quantification was performed blindly. Antibody details are presented in Table S2.

### 2.18. Expression of Lactic Acid, Pyruvate, and Cytokines in Tumors

Tumor tissues from the control and CuB groups were harvested and homogenized, and the supernatant was collected by means of centrifugation. The LA and PA concentrations in the supernatant were detected using a reagent kit. In addition, fresh tumor tissues were mixed with 0.9 mL pre-cooled PBS at a ratio of 1:9 (*w*/*v*) and homogenized on ice. The homogenate was centrifuged at 2000 rpm at 4 °C for 20 min, and the supernatant was placed on ice for subsequent detection. The pH values of tumor suspensions from mice in different treatment groups were measured using a precision pH meter (Hangzhou Qiwei Instrument Co., Ltd., Hangzhou, China), which was calibrated with standard buffer solutions (pHs 4.00, 6.86, 9.18) according to the instruction manual before detection. The levels of cytokines (i.e., iNOS, TNF-α, INF-γ, Arg1, IL-4, and IL-10) in mouse tumor lysate were detected using ELISA kits.

### 2.19. Metabolomic Analysis

For the metabolomic analysis, 30 mg of sample was mixed with 600 μL of pre-cooled 80% (*v*/*v*) methanol containing two steel balls. The mixture was pre-cooled at −40 °C, ground for 2 min, sonicated in an ice-water bath, and then incubated at −40 °C overnight. After centrifugation (12,000 rpm, 4 °C, 20 min), 150 μL of the supernatant was stored at −80 °C for subsequent LC-MS analysis. Another 150 μL of the supernatant was dried and derivatized with 80 μL of methoxyamine hydrochloride (15 mg/mL in pyridine) at 37 °C for 60 min, followed by the addition of 50 μL of BSTFA, 20 μL of n-hexane, and 10 μL of internal standards (C8-C24 prepared in chloroform); the mixture was reacted at 70 °C for 60 min and then left at room temperature for 30 min. GC-MS was used for the metabolic profiling of energy metabolites (including those related to glycolysis, the tricarboxylic acid cycle, and amino acids). Quality control (QC) samples were prepared by mixing equal volumes of extracts from all samples. Raw data were processed using XCMS v4.5.1 (for baseline filtering, peak detection, etc.) and MS-DIAL for the deconvolution analysis. Compounds were identified based on their retention time, mass, fragments, and isotopes, combined with the HMDB, Lipidmaps v2.3, METLIN, and LuMet-Animal3.0 databases. Information on the internal standards and experimental reagents is provided in Table S3.

### 2.20. Western Blotting (WB)

Protein samples underwent separation via SDS-PAGE (Lablead, P0085) and were then transferred using a transfer buffer (servicebio, G2145). After being blocked for 2 h, the membrane was incubated with the primary antibody overnight at 4 °C. Before incubation with the secondary antibody, the membrane was washed with PBS. Finally, an ultrasensitive ECL chemiluminescent solution (Biorigin, BN16009) was used, and images were taken using the chemiluminescence image analysis system. β-actin was used as the internal reference, and specific information on the antibodies used is shown in Table S2.

### 2.21. In Vivo Immunotherapeutic Mechanism

On the 11th day, the mice in each group were humanely sacrificed. Tumor tissues and spleens were then collected and subjected to flow cytometry analysis. Minced tissue was ground with PBS and sieved. For lymph node dendritic cells, sieved cells were centrifuged at 1000 rpm for 10 min; the pellet was washed with PBS, resuspended in 200 μL PBS, incubated with antibodies at room temperature for 30 min, then detected on a BD LSRFortessa Cell Analyzer. For the spleen and tumor, sieved cells were centrifuged at 1500 rpm for 10 min; the pellet was resuspended in 2 mL DMEM, layered over 1 mL room-temperature Lymphoprep, and centrifuged at 800× *g* for 25 min at room temperature. The buffy coat was collected, washed with PBS, centrifuged at 1000 rpm for 10 min, incubated with antibodies at room temperature for 30 min, then detected on a BD LSRFortessa Cell Analyzer. Antibodies were diluted to half the maximum recommended concentration, as per the product instructions.

Moreover, immunofluorescence staining was carried out on the tumor tissues to determine the expression levels of CRT and HMGB1. The antibodies used are listed in Table S2.

### 2.22. Transcriptomic Analysis

Libraries were sequenced on Illumina Novaseq 6000, yielding 150 bp paired-end reads. RNA integrity (Agilent 2100 Bioanalyzer, Santa Clara, CA, USA) was ensured with an RIN of ≥7.0. The libraries were then constructed using the VAHTS Universal V6 RNA-seq Library Prep Kit following the manufacturer’s instructions. The sequencing depth was approximately 40 million reads per sample to ensure sufficient sequencing coverage. Raw reads were processed with Fastp 1 to remove low-quality ones. After StringTie2 assembly, Cuffcompare enabled gene structure extension and novel transcript identification. ASprofile analyzed alternative splicing of differential transcripts/exons. SNPs/InDels were identified using SAMtools/BCFtools (details: http://samtools.sourceforge.net/mpileup.shtml (accessed on 15 March 2025)), with SnpEff annotating variant effects (e.g., amino acid changes). Subsequently, a bioinformatics analysis of the RNA-Seq data was performed using R software (v 3.2.0), in which the DESeq2 package in R was used to identify differentially expressed genes (DEGs). Hypergeometric-based enrichment analyses (GO, KEGG, Reactome, WikiPathways) in R screened significant terms, which were visualized as bar charts, chord diagrams, and bubble charts via R.

### 2.23. Statistical Analysis

The data are presented in the form of the mean ± the standard error of the mean (SEM), analyzed using GraphPad Prism 10.2.3. For comparisons of two groups, unpaired *t*-tests were used; for comparisons of multiple groups, one-way ANOVAs with Tukey’s post hoc test were used. Significance was inferred at *p* < 0.05. Normality (Shapiro–Wilk) and variance homogeneity (Levene) were tested; parametric methods were applied only when assumptions were met. Details of the specific statistical methods, *p*-values, and sample sizes (*n*) for each experiment are provided in the figures and their respective legends.

## 3. Results

### 3.1. Anti-Pancreatic Cancer Activity of CuB

**Figure 1:**
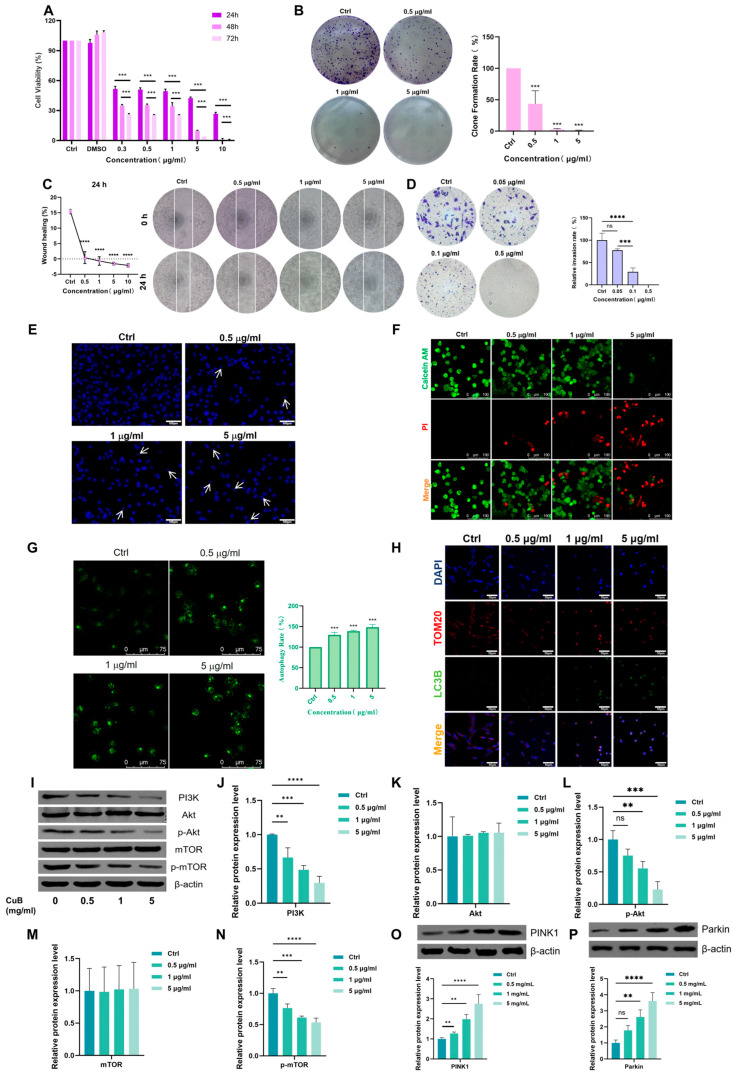
Anti-pancreatic cancer activity of CuB. (**A**) CuB has potent growth inhibitory effects on PANC-1 cells. Bars, ±standard error (SE). (**B**) Effect of CuB on colony formation. (**C**,**D**) Effects of CuB on migration and invasion. (**E**,**F**) DAPI and Calcein AM/PI staining. (**G**) Effect of CuB on autophagy. (**H**) Immunofluorescence staining of DAPI, LC3B, and TOM20. (**I**–**P**) Effects of CuB on proteins related to the PI3K/Akt/mTOR and PINK1/Parkin pathways. Bars, ±SE; ** *p* < 0.01, *** *p* < 0.001, **** *p* < 0.0001.

We explored CuB’s antitumor activities through in vitro cell experiments. To determine the effect of CuB on the proliferation of PANC-1 cells, the cells were incubated with CuB for 24 or 48 h. Our results showed that CuB significantly inhibited the proliferation of PANC-1 cells (Figure 1A) in a time- and dose-dependent manner, and the IC50 values were 1.0, 0.11, and 0.022 μg/mL for 24, 48, and 96 h. Even at low concentrations, CuB significantly inhibited the colony formation and migration abilities of PANC-1 cells (Figure 1B,C). A Transwell assay with Matrigel showed that CuB reduced the invasion ability of PANC-1 cells (Figure 1D). As shown in Figure 1E,F, CuB also induced cell death in a concentration-dependent manner, causing the cell nuclei to shrink and become brighter.

To further study the mechanism of cell death, we also examined the autophagic effect of CuB on PANC-1 cells. The number of autophagic vesicles increased significantly in cells treated with CuB (Figure 1G). Meanwhile, as the concentration of CuB increased, the intensity of the fluorescence co-localization of the autophagy protein LC3B and mitochondria (labeled by TOM20) was enhanced (Figure 1H), indicating that the ability of autophagosomes to recognize and clear damaged mitochondria improved, and the level of mitophagy increased. The results (Figure 1I–P) of WB in vitro showed that after drug administration, PI3K, *p*-mTOR, and *p*-Akt expression decreased, total mTOR and total Akt remained unchanged, and PINK1 and Parkin expression increased. Therefore, CuB may induce mitophagy through the PI3K/Akt/mTOR and PINK1/Parkin pathways.

### 3.2. CuB Induces Immunogenic Cell Death (ICD)

**Figure 2:**
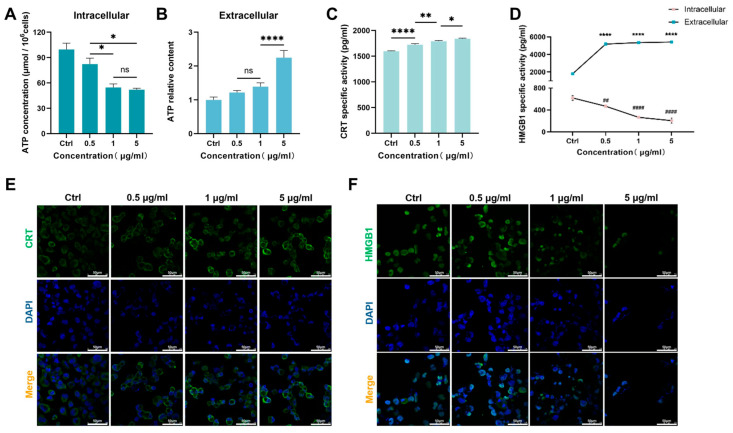
CuB induces immunogenic cell death. Effects of CuB on the intracellular (**A**) and (**B**) extracellular ATP contents in PANC-1 cells. The levels of (**C**) CRT and (**D**) HMGB1 in PANC-1 cells. (**E**) CRT and (**F**) HMGB1 immunofluorescence staining. Bars, ±SE; * *p* < 0.05, ** *p* < 0.01, **** *p* < 0.0001, ## *p* < 0.01, #### *p* < 0.0001.

ICD can directly cause cell death and trigger immune responses to combat cancer. This process is marked by the presentation of CRT on the cell surface. Additionally, it involves the secretion of mediators, including the non-histone chromatin protein HMGB1 and ATP [20,21]. We also investigated the effect of CuB on ICD. ATP is released in an autophagy-dependent manner from cells during the ICD process [22]; therefore, we detected the ATP content. Figure 2A,B shows that CuB significantly reduced intracellular ATP levels but increased ATP release. Additionally, CuB promoted the release of HMGB1 and the exposure of CRT, while the intracellular expression of HMGB1 was decreased (Figure 2C–F). This suggests that during the action of CuB, an ICD response occurred in the tumor cells, which enhanced the antitumor immune response.

### 3.3. Cucurbitacin B-Mediated Inhibition of Glycolysis

**Figure 3:**
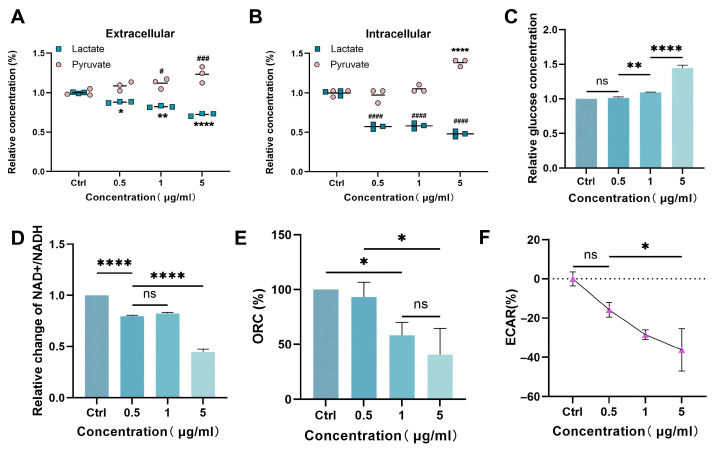
Cucurbitacin B-mediated inhibition of glycolysis. (**A**) Extracellular and (**B**) intracellular LA and PA contents in PANC-1 cells. (**C**) Intracellular glucose concentration in PANC-1 cells. NAD+/NADH ratio (**D**), ORC (**E**), and ECAR (**F**) in PANC-1 cells under different concentrations of CuB. Bars, ±SE; * *p* < 0.05, ** *p* < 0.01, **** *p* < 0.0001, # *p* < 0.05, ### *p* < 0.001, #### *p* < 0.0001.

To explore the impacts of CuB on cellular glucose metabolism, we initially measured the levels of both intracellular and extracellular LA and PA. The findings indicated that following drug administration, the concentrations of both intracellular and extracellular PA rose, whereas those of LA declined (Figure 3A,B), indicating that the glycolytic pathway might have been inhibited. The accumulation of PA led to the inhibition of glucose metabolism, resulting in a decrease in glucose consumption (Figure 3C). Additionally, we found that CuB decreased the NAD^+^/NADH ratio, along with both the OCR and ECAR (Figure 3D–F), indicating that the cellular oxidative phosphorylation metabolic pathway was also inhibited.

**Figure 4:**
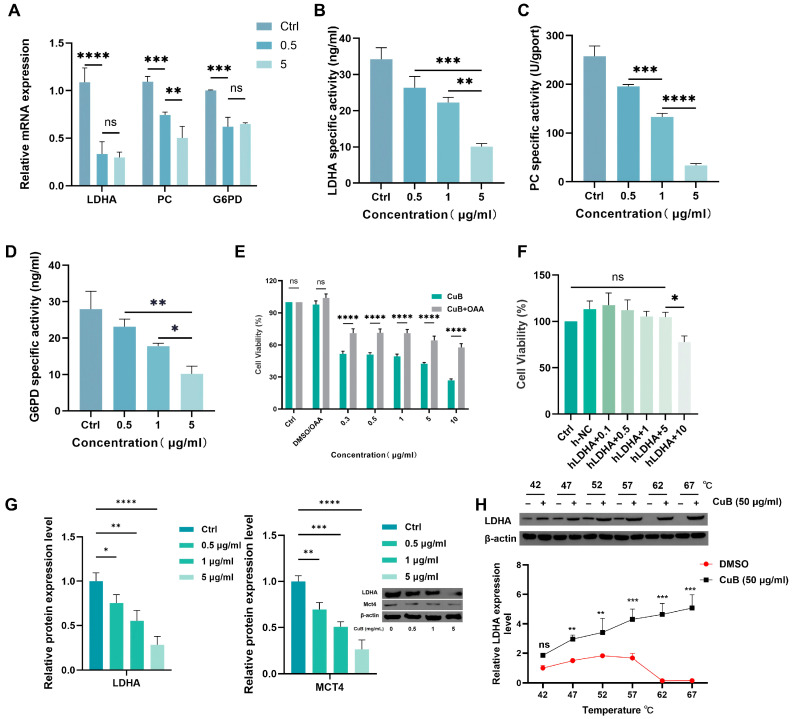
The mechanism by which CuB inhibits glycolysis. (**A**) The transcription levels and (**B**–**D**) ELISA quantitative analysis results of three enzymes in PANC-1 cells treated with CuB. (**E**) The viability of CuB-treated PANC-1 cells with/without OAA. (**F**) The cell viability of CuB-treated PANC-1 cells after LDHA overexpression. (**G**) Protein expression of LDHA and MCT4 in PANC-1 cells under CuB. (**H**) CETSA. Bars, ±SE; * *p* < 0.05, ** *p* < 0.01, *** *p* < 0.001, **** *p* < 0.0001.

To further clarify the mechanism of CuB’s effect on glucose metabolism, we detected the transcription of LDHA (a key enzyme in glycolysis), PC (a key enzyme in the TAC), and G6PD (a key enzyme in the PPP). The transcription levels of all three enzymes were downregulated after drug administration (Figure 4A), which was also confirmed by the ELISA results (Figure 4B–D). Among them, the activities of PC and LDHA were more significantly affected by CuB than were those of G6PD. Pyruvate reacts with CO_2_ in a carboxylation reaction catalyzed by PC to generate oxaloacetate (OAA), which then enters the TCA cycle pathway. Our experiments demonstrated that CuB significantly inhibited PC activity (Figure 4C). Thus, we examined the effect on PANC-1 cells with added OAA (2 mM). The results indicated that the addition of OAA (2 mM) only slightly rescued the growth of PANC-1 cells treated with CuB (Figure 4E). Moreover, the inhibitory effect of CuB on PANC-1 cells with LDHA overexpression (Figure S1) was significantly attenuated (Figure 4F). This indicated that CuB mainly restrained the expression of LDHA enzyme, suppressing the glycolytic pathway.

The WB experiment also confirmed that the LDHA level decreased in the CuB-treated group (Figure 4G). In addition, we investigated MCT4 expression by means of WB. The downregulation of MCT4 expression (Figure 4G) indicated that CuB also reduced lactate efflux. To further determine whether LDHA is the target of CuB, we performed a CETSA (Figure 4H). The LDHA activity in the DMSO group decreased with an increase in temperature but was retained after treatment with CuB. These results indicate that CuB may influence the glucose metabolism reprogramming of cells by inhibiting the activities of enzymes related to glucose metabolism in PANC-1 cells—particularly the activity of LDHA, a key enzyme in glycolysis—thereby reducing lactate production and ATP generation and consequently suppressing aerobic glycolysis and growth in PANC-1 cells.

### 3.4. In Vivo Antitumor Effect of CuB

**Figure 5:**
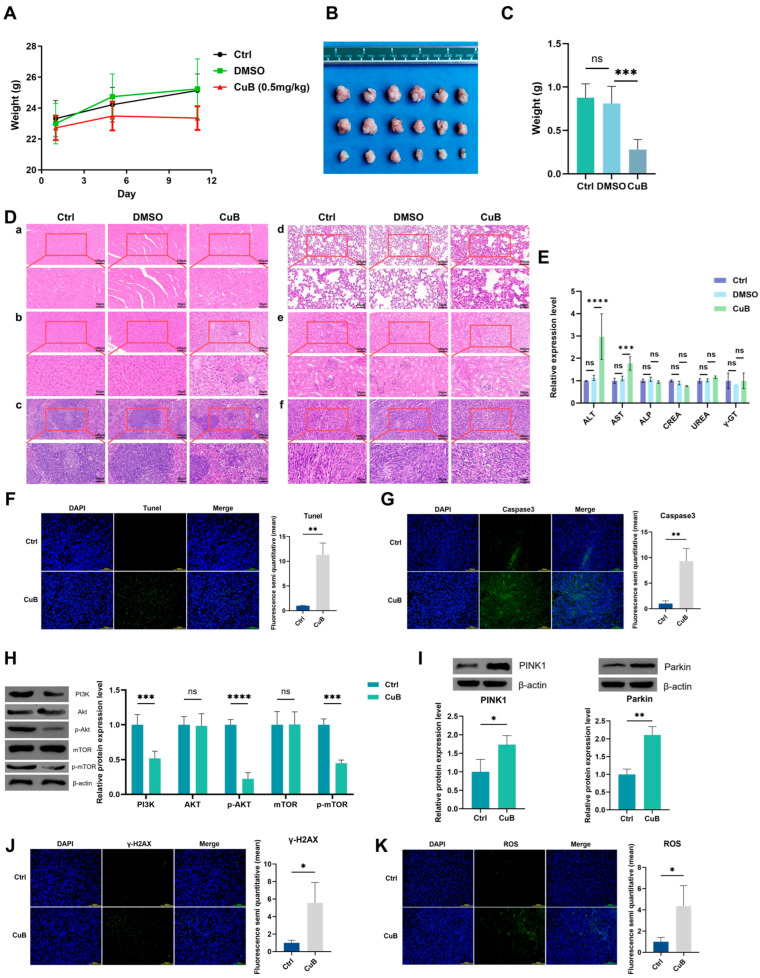
In vivo antitumor effect of CuB. (**A**) Weights of mice (*n* = 6). Volumes (**B**) and weights (**C**) of tumors at the end of treatment (*n* = 6). (**D**) HE staining images of the heart (**a**), liver (**b**), spleen (**c**), lung (**d**), kidney (**e**), and tumors (**f**) of mice at the end of different treatments. (**E**) Blood biochemical index analysis; *n* = 6. IF staining of TUNEL (**F**) and caspase-3 (**G**) in tumor tissue. (**H**,**I**) WB analysis of proteins related to the PI3K/Akt/mTOR and PINK1/Parkin signaling pathways. γ-H2AX staining (**J**) and ROS staining (**K**) in tumor tissue. *n* = 3; bars, ±SE; statistical significance determined via unpaired two-tailed Student’s *t*-test; * *p* < 0.05, ** *p* < 0.01, *** *p* < 0.001, **** *p* < 0.0001.

To explore the in vivo anticancer effect of CuB, we established a PANC02 tumor-bearing mouse model and examined the changes in tumor volume and weight in three different treatment groups (saline, 0.1% DMSO, and CuB). First, the in vivo safety of the CuB treatment was affirmed by tracking body weight, assessing blood biochemical indices, and conducting HE staining. The experimental data indicated that CuB did not cause a significant reduction in the body weight of the mice (Figure 5A). However, compared with those for the other groups, the rate of weight gain was decreased, which might be related to the regulatory effect of CuB on mouse metabolism. It also caused no evident inflammation or damage in the major organs of the mice (including heart, spleen, lung, and kidneys), but some inflammation occurred in the liver (Figure 5E). Furthermore, there was a significant difference in the growth of tumors (Figure 5B,C) among the CuB-treated group, the saline group, and the 0.1% DMSO group. H&E staining (Figure 5D) showed that tumors in CuB-treated mice exhibited pronounced cellular damage, including widespread structural disintegration and nuclear loss, underscoring the remarkable antitumor potency of CuB. To further assess tumor cell apoptosis, TUNEL and caspase-3 assays were performed. Figure 5F,G demonstrate marked increases in TUNEL-positive cells and caspase-3 expression in the CuB group compared with the controls, confirming CuB’s capacity to trigger apoptotic pathways in tumor cells.

We further detected the expression of mTOR, *p*-mTOR, PI3K, Akt, *p*-Akt, PINK1, and Parkin in tumor tissues via WB. The results (Figure 5H,I) were consistent with those of the in vitro experiments. CuB autophagy induction is associated with the induction of ROS and the promotion of DNA damage [23,24,25]. Therefore, we also detected the levels of γ-H2AX and ROS in tumor tissues by means of immunofluorescence. In this study, as shown in Figure 5J,K, the fluorescence of both γ-H2AX and ROS in the CuB treatment group was enhanced.

### 3.5. In Vivo Effect of CuB on Tumor Metabolism

**Figure 6:**
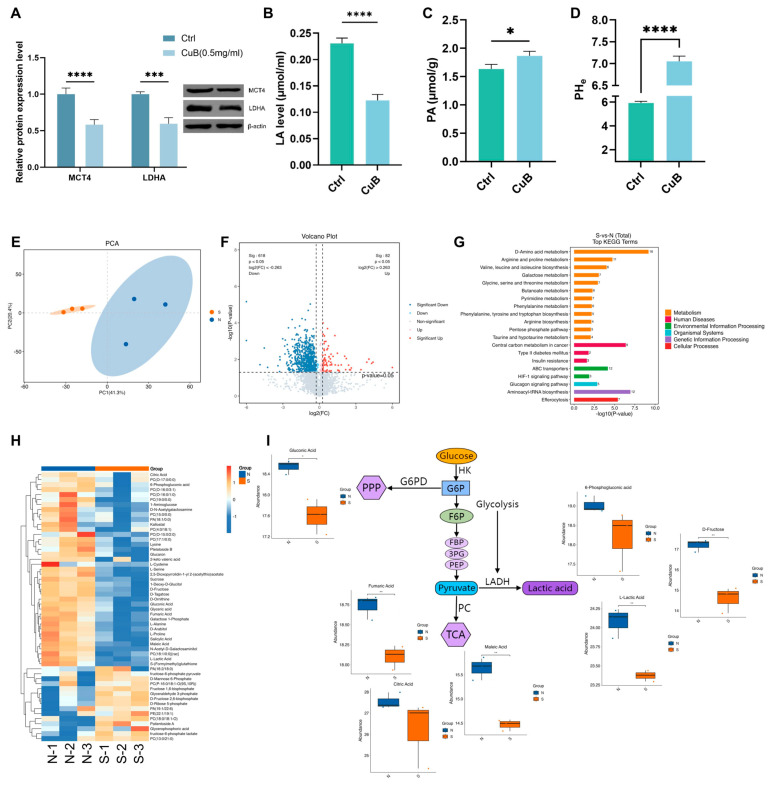
In vivo metabolism effect of CuB. (**A**) Protein expressions of LDHA and MCT4 in tumor tissue (*n* = 3). Concentrations of LA (*n* = 6) (**B**) and PA (*n* = 3) (**C**). (**D**) pH values in the supernatant of mouse tumor tissue (*n* = 3). (**E**) PCA of metabolites in tumors from mice (*n* = 3). Volcano plot (**F**) and KEGG enrichment analysis (**G**) of all DEMs between N (control group) and S (CuB-treated group). (**H**) Clustering heatmap of differentially expressed metabolites related to glucose metabolism. (**I**) Schematic of CuB-induced changes in metabolic pathways. Bars, ±SE; statistical significance determined via unpaired two-tailed Student’s *t*-test; * *p* < 0.05, ** *p* < 0.01, *** *p* < 0.001, **** *p* < 0.0001.

In pancreatic cancer, high glycolytic rates and the accumulation of LA contribute to the creation of the acidic pericellular TME and activate immunosuppressive pathways [26]. To elucidate the potential mechanism of CuB in regulating glycolytic metabolism and reversing an immunosuppressive TME, we first investigated the glycolysis-related enzyme levels in vivo after CuB treatment. Consistent with the in vitro results, in tumor tissues, the expression levels of LDHA and MCT4 in the CuB treatment group were decreased (Figure 6A), the content of LA was reduced, and the content of PA was increased (Figure 6B,C). Studies have shown that MCTs, especially MCT4, which transports LA out of the cell, are important for pH regulation [27]. In this study, we found that the pH value of tumor tissues in mice significantly increased to 7.05 (Figure 6D), indicating that the acidic microenvironment was improved.

To further elucidate the anticancer effects of CuB, we conducted a metabolomics analysis. The principal component analysis (PCA) results indicated marked disparities between the normal saline group and the CuB-treated group (Figure 6E). Among all the detected differential metabolites, 214 were downregulated, while 51 were upregulated (Figure 6F). The expression of the metabolites after CuB treatment is shown in a clustered heatmap. The metabolomics analysis combined with KEGG pathway annotation revealed that the differentially expressed metabolites (DEMs) were predominantly enriched in three major metabolic pathways: amino acid metabolism, carbohydrate metabolism, and lipid metabolism (Figure 6G). Building upon our metabolomic investigation, we systematically characterized the DEMs associated with glycolytic pathways and glucose homeostasis. The clustered heatmap (Figure 6H) demonstrates that glucose metabolites like L-LA were substantially diminished in the CuB-treated group. The levels of relevant metabolites of the PPP pathway, TCA cycle, and glycolysis pathway were notably decreased (Figure 6I), suggesting significant suppression of glucose metabolism.

### 3.6. In Vivo Effect of CuB on Tumor Immune System

**Figure 7:**
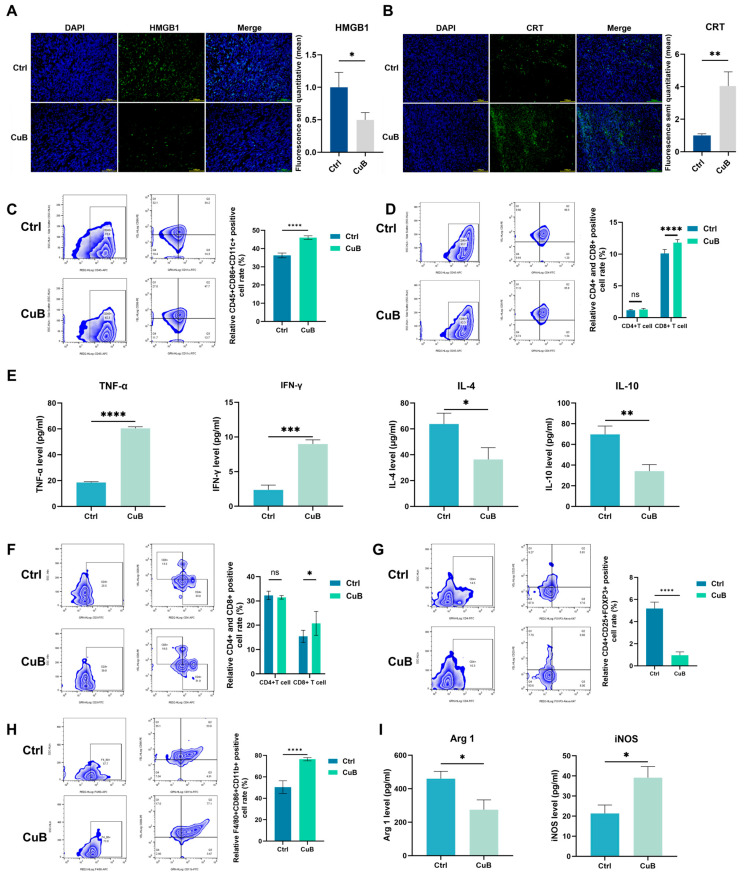
In vivo effect of CuB on tumor immune system. Immunofluorescence staining and quantitative fluorescence analysis of HMGB1 (**A**) and CRT (**B**). DC maturation in tumor tissues (**C**) and CD8+ T cells and CD4+ cells in the spleen (**D**). (**E**) Expression levels of TNF-α, IFN-γ, IL-4, and IL-10. CD8+ T cells and CD4+ T cells (**F**), Tregs (**G**), and M1-polarization macrophages (**H**) in tumor tissues. (**I**) Expression of M1 and M2 polarization markers. *n* = 3; bars, ±SE; statistical significance determined via unpaired two-tailed Student’s *t*-test; * *p* < 0.05, ** *p* < 0.01, *** *p* < 0.001, **** *p* < 0.0001.

In vitro studies have shown that CuB can induce ICD. In vivo, immunofluorescence staining (Figure 7A,B) showed that treatment with CuB led to significantly higher exposure of CRT and lower exposure of HMGB1 than those in the saline group, which means that it had excellent ICD induction performance. We further examined the maturation of DCs. The maturity of DCs in the CuB-treated group was significantly increased, accounting for 46.04% (Figure 7C). Moreover, we noted that after CuB treatment, the proportion of CD8+ T cells among T lymphocytes infiltrating the mouse spleen increased by 1.23-fold. Meanwhile, the CD4+ T-cell proportion showed no marked disparity (Figure 7D).

Our results show that CuB significantly inhibits the secretion of LA. A reduction in LA secretion could decrease the levels of anti-inflammatory cytokines while increasing the levels of pro-inflammatory cytokines [28]. Therefore, we tested the relevant factors in the supernatant; the ELISA results (Figure 7E) showed increased expression levels of TNF-α and INF-γ (3.20- and 3.79-fold, respectively) and decreased levels of IL-4 and IL-10. This indicates that CuB exerts an in vivo effect of inducing inflammatory factors that are closely related to the activation of systemic immunity [29]. Among them, cytokines such as IFN-γ and TNF-α are key biomarkers produced by tumor-infiltrating T cells [30]. Then, we examined the proportions of CD8+ T cells and CD4+ T cells among the tumor-infiltrating T cells. As illustrated in Figure 7F, the infiltration of CD8+ T lymphocytes increased 1.35-fold after treatment with CuB, while CD4+ T-cell populations remained statistically unchanged. Additionally, Tregs play a key role in mediating immunosuppression within the TME by inhibiting both T-cell expansion and effector cytokine secretion. In our results, the number of Treg cells in the CuB treatment group was significantly reduced (Figure 7G). Notably, CuB treatment triggered macrophage polarization toward the M1 phenotype (Figure 7H), as evidenced by upregulated M1-specific markers and concomitant downregulation of M2-associated markers (Figure 7I).

The above results demonstrate that CuB improves tumor glycolytic metabolism by inhibiting the expression of LDHA, thereby reducing LA production, remodeling systemic immunity, and increasing the infiltration of antitumor immune cells.

### 3.7. Transcriptome Analysis

**Figure 8:**
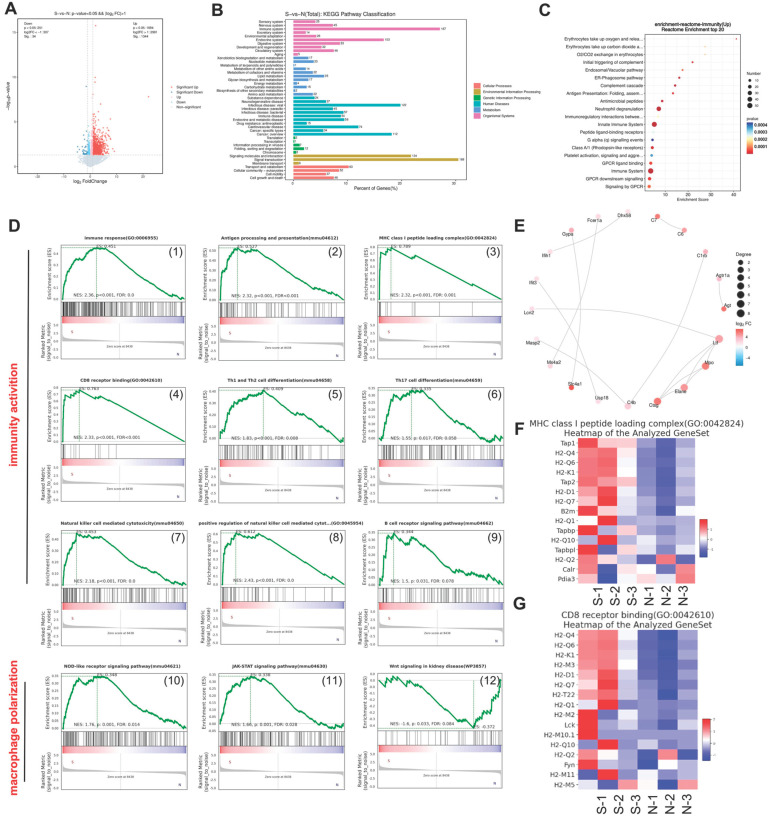
RNA-Seq analysis of tumor tissues receiving CuB. A volcano plot (**A**) and KEGG enrichment analysis (**B**) of all DEGs between N (control group) and S (CuB-treated group). (**C**) The results of the Reactome enrichment analysis based on the immunomodulatory pathways. (**D**) The enrichment of differentially expressed genes was investigated by means of GSEA ((1)–(9) are immune activation-related pathways, and (10)–(12) are macrophage polarization-related pathways.). (**E**) The PPI network of differentially expressed genes. Clustering heatmaps for “MHC class I peptide loading complex” (**F**) and “CD8 receptor binding pathway” (**G**).

Abnormal tumor metabolism—such as glycolysis—and the accumulation of LA in tumors play important roles in establishing an immunosuppressive TME [30]. Therefore, we used RNA-Seq to explore the mechanisms by which CuB regulates glycolysis and activates the immune response. As shown in the volcano plot in Figure 8A, we detected 1378 DEGs. Upregulated genes (1344) are indicated in red, while downregulated genes (34) are shown in blue. A KEGG pathway enrichment analysis was conducted on the DEGs. As shown in Figure 8B, the DEGs in organismal systems were mainly enriched in the immune system. The subsequent Reactome pathway analysis of immunomodulatory pathways is presented in Figure 8C. Furthermore, large immunity-associated pathways, including antigen presentation, activation of multiple immune cells, and macrophage polarization, were found via GSEA to be enriched in the CuB group (Figure 8D). The top 30 genes in the PPI network showed that most hub genes were related to immunity (Figure 8E). In particular, CuB treatment significantly activated the “MHC class I peptide loading complex” and “CD8 receptor binding pathway” (Figure 8F,G) and increased the number of CD8^+^ T cells (Figure 7F). In summary, immune function was significantly activated after CuB treatment, including antigen presentation and the activation of immune cells to enhance antitumor immune responses.

## 4. Discussion

**Figure 9:**
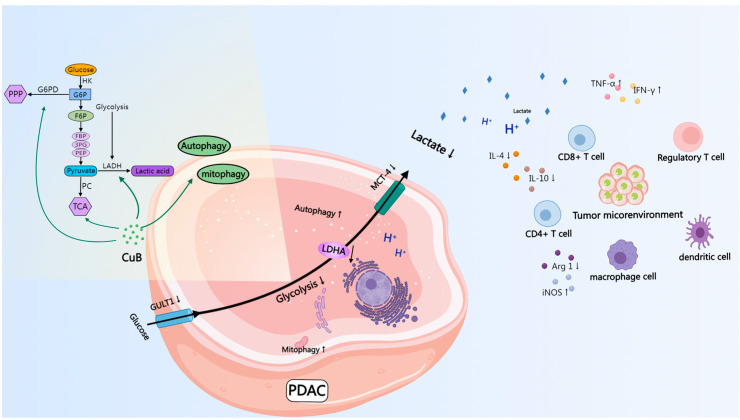
An illustration of CuB’s mechanism of action. (Created with MedPeer (medpeer.cn).)

CuB exhibits broad-spectrum antiproliferative activity against multiple cancer cell types. Its mechanisms include inducing cell cycle arrest [31], triggering autophagy [23], hindering cell migration and invasion [32], and suppressing angiogenesis [33]. Moreover, CuB exhibits immune-enhancing properties by promoting the M1 polarization of macrophages [34] and enhancing dendritic cell differentiation [35]. Despite its promising effects, the full mechanism of CuB’s action remains elusive. Our study revealed that CuB initiates mitophagy in PDAC cells via the PI3K/Akt/mTOR and PINK1/Parkin pathways. Additionally, it reduces lactate secretion by downregulating LDHA expression, which hinders tumor glycolysis and boosts the antitumor immune response. This multifaceted approach underscores CuB’s potential as a potent anticancer agent (Figure 9).

This study revealed that CuB exerts a multifaceted influence, significantly inhibiting not only the proliferative abilities of PANC-1 cells but also their abilities to form colonies, invade, and migrate. Our finding that CuB significantly inhibits the PI3K/Akt/mTOR pathway, a well-known negative regulator of autophagy, is consistent with the findings of prior research [36,37]. The PI3K/Akt/mTOR pathway is activated after administration of the drug. Furthermore, TFAM, a key regulatory factor of mitochondrial function, shows a reduction in mitochondria that reflects mitochondrial structural damage and degradation, consistent with the occurrence of mitophagy [38]. We demonstrated that TFAM expression decreases with increasing CuB concentration (Figure S3A), while the ratio of intracellular to cytoplasmic mtDNA increases, indicating enhanced mitophagy and the release of mtDNA into the cytoplasm (Figure S3B). CuB activates the PINK1/Parkin pathway, recognized as the core machinery for mitophagy [39]. For the first time, we demonstrated that CuB induces selective autophagy, specifically mitophagy, through the PINK1/Parkin pathway. This dual regulation not only promotes the clearance of damaged mitochondria and the release of mtDNA, but also disrupts the energy metabolism balance of tumor cells, contributing to the inhibition of tumor growth.

CuB also shows a significant inhibitory effect on glycolysis in PDAC cells, crucial for breaking the “Warburg effect” on which tumor cells depend. In the CuB-treated group, indicators related to glucose metabolism—glucose consumption, the NAD+/NADH ratio, the OCR, and the ECAR—were reduced (Figure 3D–F). Simultaneously, the LA content decreased, while PA accumulated. Further study revealed that this phenomenon is mainly due to CuB suppressing the expression of LDHA, a key enzyme in glycolysis that catalyzes the conversion of PA to LA. A CETSA experiment confirmed LDHA as a direct target of CuB. Reduced LDHA activity not only impairs glycolytic energy supply but also decreases extracellular LA export through MCT4, alleviating tumor microenvironment (TME) acidosis (Figure 6D). These results form an extension of research by Li et al. [40], who linked CuB’s inhibition of glycolytic enzymes (HK, PK) to Akt/mTOR regulation, by identifying LDHA as a critical downstream effector.

In addition, the CuB-treated group exhibited an increase in extracellular ATP content, CRT exposure, and HMGB1 production inhibition—hallmarks of ICD. The DAMPs released during ICD can be phagocytosed by antigen-presenting cells (APCs), such as dendritic cells (DCs). The maturation of DCs has been demonstrated as a crucial process in initiating immune responses through facilitating antigen presentation and the infiltration of cytotoxic T lymphocytes [41]. In this study, we examined immune cells and cytokines in CuB-treated mice. The results showed that CuB promoted DC maturation, increased the number of CD8+ T cells, reduced the number of Tregs, and increased the proportion of M1 macrophages. Additionally, the increase in inflammatory cytokine secretion, decrease in anti-inflammatory cytokine secretion, increase in M1 polarization markers, and decrease in M2 polarization markers suggest that CuB had a certain improvement effect on the immunosuppressive TME. Notably, the level of LA—a known suppressor of T-cell function and inducer of M2 polarization [42,43]—is reduced by CuB, further amplifying immune activation.

RNA sequencing results showed that the upregulated genes were enriched in immune-related pathways, indicating significant activation of immune function after CuB treatment. In particular, pathways related to antigen presentation were significantly activated. Enhanced IFN-γ secretion likely drives increased MHC-I expression, facilitating antigen presentation to CD8+ T cells [44] and mitigating immune escape. IF and WB results also confirmed that MHC-I expression increased after CuB treatment (Figure S4). These transcriptomic findings align with phenotypic changes in the TME (Figure 7D–F), reinforcing CuB’s ability to coordinate metabolic disruption with immune activation.

Compared with standard PDAC therapies, CuB exhibits distinct mechanisms of action. Gemcitabine (a nucleoside analog) and FOLFIRINOX (a combination of 5-fluorouracil, leucovorin, irinotecan, and oxaliplatin) primarily target DNA synthesis or cell division but are limited by drug resistance and systemic toxicity [45]. In contrast, CuB acts through unique pathways: it directly targets glycolysis via LDHA, exploiting PDAC’s metabolic dependency, whereas gemcitabine inhibits DNA replication; it reshapes the TME to enhance antitumor immunity, a feature not prominent in FOLFIRINOX, potentially synergizing with immunotherapies. It is worth noting that CuB-induced PINK1/Parkin-mediated mitophagy accelerates mitochondrial clearance and disrupts the energy metabolism balance, as evidenced by the reduced level of oxidative phosphorylation (Figure 3E). Moreover, abnormal mitophagy may lead to the release into the cytoplasm or extracellular space of mtDNA or mitochondrial proteins, which can activate the cGAS-STING pathway or be recognized by immune cells as damage-associated molecular patterns (DAMPs). This process promotes antigen presentation and antitumor immune responses, suggesting that CuB could serve as an adjuvant for PDAC immunotherapy—for instance, by enhancing the efficacy of PD-1/PD-L1 inhibitors and improving the “immune desert” TME of PDAC. This also represents a direction for our further research.

It is worth noting that in our experiments, we found that even a low concentration of CuB can cause liver damage in mice, limiting its clinical application. Currently, a growing number of nanomaterials, such as Metal–Organic Frameworks (MOFs), exosomes, liposomes, etc., are being utilized as carriers for natural drugs. These carriers, when modified, exhibit targeting and controlled release capabilities. Among them, MOFs, composed of metal ions and organic ligands, boast a high drug loading capacity. When targeted modifications are applied, they can enhance efficacy and reduce toxicity. Hence, utilizing MOFs as carriers for the targeted delivery of CuB represents a direction for our future research.

## 5. Conclusions

In conclusion, CuB inhibits PDAC through a coordinated interplay of autophagy induction, metabolic disruption, and TME remodeling. Its unique mechanism of action, distinct from those of standard therapies, highlights its potential as a multi-target antitumor agent, with further research into delivery systems poised to address its clinical limitations. Certainly, there is still room for improvement in this article. For instance, the PANC-02 cell line model was used to establish tumor-bearing mice. Although this model can better simulate the tumor-immune interactions in immunocompetent mice, due to differences in the gene mutation spectrum compared with human PDAC, it cannot be used as a direct basis for translational therapy in human PDAC, but only provides a foundation for subsequent verification in more precise models (such as mouse models with driver gene mutations like KRAS and TP53).

## Data Availability

Data will be made available on request.

## Supplementary Materials

The following supporting information can be downloaded at: https://www.mdpi.com/article/10.3390/nu17172809/s1, Figure S1: The transcription levels of LDHA in PANC-1 cells; Figure S2: The nuclear magnetic resonance (NMR) results of CuB; Figure S3: (A) Immunofluorescence staining of DAPI, TFAM, and TOM20. (B) The transcription levels of mtDNA in PANC-02 cells treated with CuB (1 μg/mL); Figure S4: The IF staining (A) and protein expression of MHC-I in tumor tissue (*n* = 3); Table S1: Primers used in this study; Table S2: Antibodies used in this study.(IF, WB, Flow); Table S3: Reagent Information.

## Abbreviations

CuB, Cucurbitacin B; DAMPs, damage-associated molecular patterns; ECAR, Extracellular Acidification Rate; GSEA, gene set enrichment analysis; GT, Gamma-Glutamyl Transferase; ICD, Immunogenic Cell Death; LA, lactic acid; OAA, oxaloacetate; OCR, Oxygen Consumption Rate; OXPHOS, oxidative phosphorylation; PA, pyruvic acid; ROS, reactive oxygen species; TCA, tricarboxylic acid cycle; TIME, tumor immunosuppressive microenvironment; TME, tumor microenvironment; TNF-α, Tumor Necrosis Factor-alpha.
