# Supplementary material for: Cucurbitacin B from Cucurbitaceae Plants: Treating Pancreatic Cancer via Inducing Mitophagy, Inhibiting Glycolysis, and Enhancing Immune Function
Source: Nutrients. 2025 Aug 29;17(17):2809. doi: 10.3390/nu17172809 (PMC12430665; doi:10.3390/nu17172809)
Supplement: Supplementary file 1 [file nutrients-17-02809-s001.zip › nutrients-3787820-supplementary.pdf]

Table S1. Primers used in this study.

Name	Assay	Sequence
GAPDH-F[a)]	RT-PCR	GCACCGTCAAGGCTGAGAAC
GAPDH-R	RT-PCR	TGGTGAAGACGCCAGTGGA
LDHA-F	RT-PCR	TTGTTGGGGTTGGTGCTGTTG
LDHA-R	RT-PCR	GGCTGCCATGTTGGAGATCC
PC-F	RT-PCR	GGGTCCGGTTTATTGG
PC-R	RT-PCR	GGAAGCCGTAGGTGTTG
G6PD-F	RT-PCR	CCAACCGCCTCTTCTACCTG
G6PD-R	RT-PCR	AGGGCTTCTCCACGATGATG
GAPDH-F[b)]	RT-PCR	TGTGTCCGTCGTGGATCTGA
GAPDH-R	RT-PCR	TTGCTGTTGAAGTCGCAGGAG
mtDNA-F	RT-PCR	CCTCCCATTGATTATCGCCGCCCTTGC
mtDNA-R	RT-PCR	GTCTGGGTCTCCTAGTAGGTCTGGGAA

[a)]F, forward; R, reverse.[b)]From this line onwards, the species referred to is mouse.

Table S2. Antibodies used in this study.(IF, WB, Flow)

Antigen	Catalog Number	Supplier
β-actin	ab8227	abcam
CRT	ab92516	abcam
HMGB1	A19529	abcam
TOM20	ab56783	abcam
LC3B	18725-1-AP	proteintech
TFAM	ab307302	abcam

PI3K	20584-1-AP	proteintech
Akt	10176-2-AP	proteintech
p-Akt	66444-1-Ig	proteintech
mTOR	28273-1-AP	proteintech
p-mTOR	67778-1-Ig	proteintech
LDHA	19987-1-AP	proteintech
MCT4	22787-1-AP	proteintech
PINK1	23274-1-AP	proteintech
Parkin	66674-1-Ig	proteintech
γ -H2AX	2F3	Biolegend
MHC-I	ab281902	abcam
Caspase-3	25128-1-AP	proteintech
ACP anti-CD45	982304	Biolegend
FITC anti-CD11c	117305	Biolegend
PE anti-CD86	159203	Biolegend
FITC anti-CD4	130308	Biolegend
PE anti-CD8	140408	Biolegend
FITC anti-CD3	100203	Biolegend
APC anti-CD4	116013	Biolegend
APC anti-F4/80	123115	Biolegend
FITC anti-CD11b	101205	Biolegend

Table S3. Reagent Information

Reagent Name	**Catalog Number**	**Supplier**	Purity
Methanol	A452-4	Fisher	HPLC, 99.9%
Acetonitrile	A998-4	Fisher	HPLC, 99.95%
Formic Acid	A117-50	Fisher	HPLC, 99.0%
Water	——	Wahaha	/

L-2-Chlorophenylalanine (Mixed Internal Standard)	C2001	Hengchuang	HPLC, 98.0%
Succinic - d4 Acid (Mixed Internal Standard)	293075-1G	Sigma	HPLC, 98.0%
L-Valine - d8 (Mixed Internal Standard)	HY-I1124	Haoyuan	HPLC, 98.0%
Cholic Acid - D4 (Mixed Internal Standard)	S22155-50mg	Yuanye	HPLC, 98.0%
D-Luciferin Free Acid (Mixed Internal Standard)	S19260-100mg	Yuanye	BR grade, 99.0%
Chloroform	G75915B	Greagent	AR grade; 99.0%
n-Hexane	4.011518.0500	CNW	HPLC, 98.5%
Pyridine	P141169-1L	Aladdin	HPLC, 99.0%
BSTFA	B0830-25ml	TCI	0.95
O-Methylhydroxylamine Hydrochloride (97%)	M813479-25g	Macklin	0.98
Methyl Caprylate Standard	G162300	Dr. Ehrenstorfeh	HPLC, 99.0%
Methyl Nonanoate (C9:0) Standard	N-9M-AU4-B	NU-chek	HPLC, 99.0%
Methyl Decanoate (C10:0) Standard	N-10M-A18-D	NU-chek	HPLC, 99.0%
Methyl Dodecanoate/Laurate (C12:0) Standard	N-12M-AU15-D	NU-chek	HPLC, 99.0%
Methyl Tetradecanoate/Myristate (C14:0) Standard	N-14M-A24-E	NU-chek	HPLC, 99.0%
Methyl Hexadecanoate/Palmitate (C16:0) Standard	G161798	Dr. Ehrenstorfeh	HPLC, 99.0%
Methyl Octadecanoate/Stearate (C18:0) Standard	N-18M-O9-C	NU-chek	HPLC, 99.0%
Methyl Eicosanoate/Arachidate (C20:0) Standard	N-20M-J27-E	NU-chek	HPLC, 99.0%
Methyl Docosanoate/Behenate (C22:0) Standard	N-22M-JY30-E	NU-chek	HPLC, 99.0%
Methyl Tetracosanoate/Lignocerate (C24:0) Standard	N-24M-S6-A	NU-chek	HPLC, 99.0%

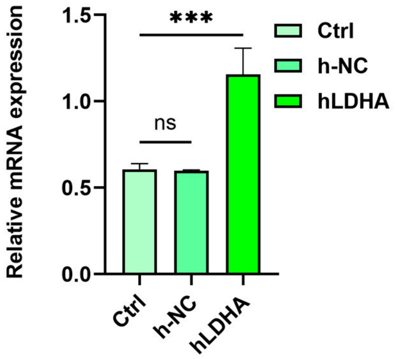

Fig. S1. The transcription levels of LDHA in PANC-1 cells. Bars, ± SE; ***p＜0.001.

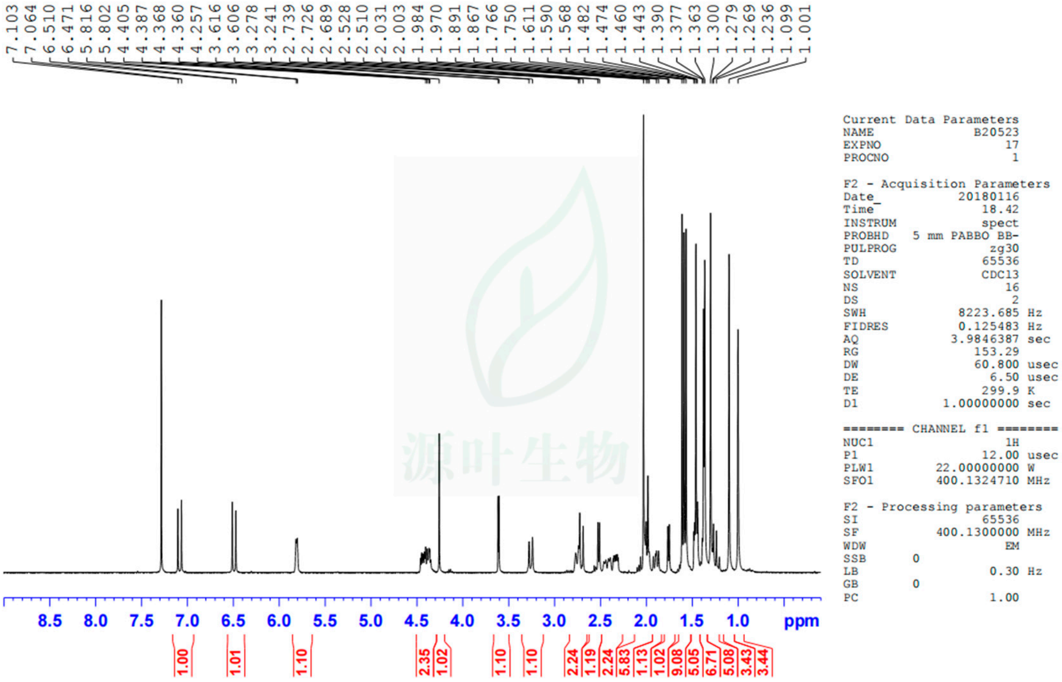

Fig. S2. The nuclear magnetic resonance (NMR) results of CuB.

(https://www.shyuanye.com/goods-B20523.html)

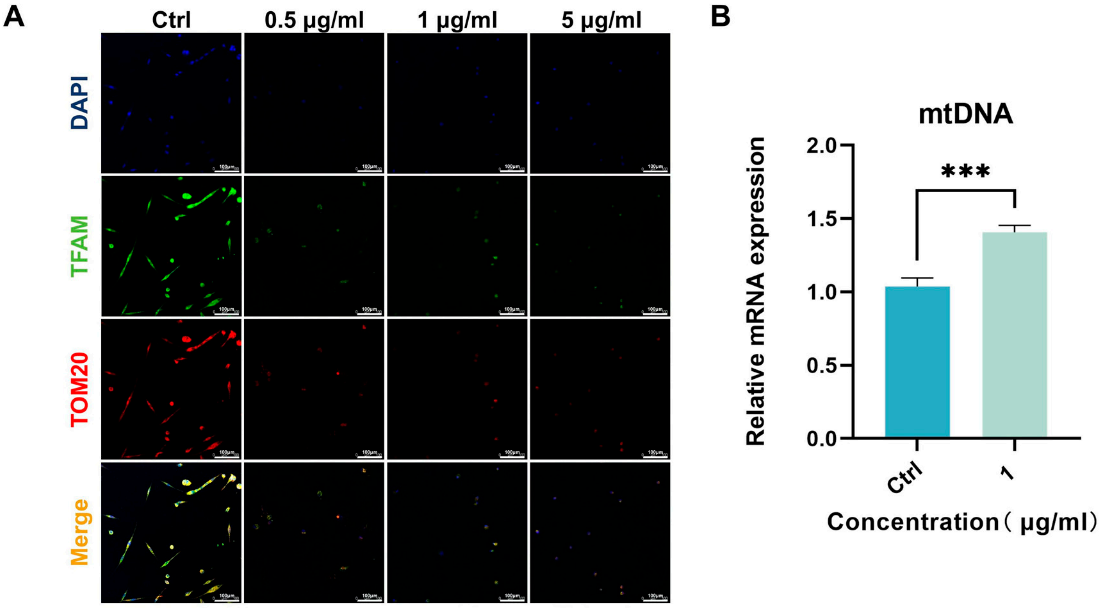

Fig. S3. (A) Immunofluorescence staining of DAPI, TFAM, and TOM20. (B) The transcription levels of mtDNA in PANC-02 cells treated with CuB (1 μg/ml). Bars, ± SE; Statistical significance was determined by an unpaired two-tailed Student's t-test; ***p<0.001.

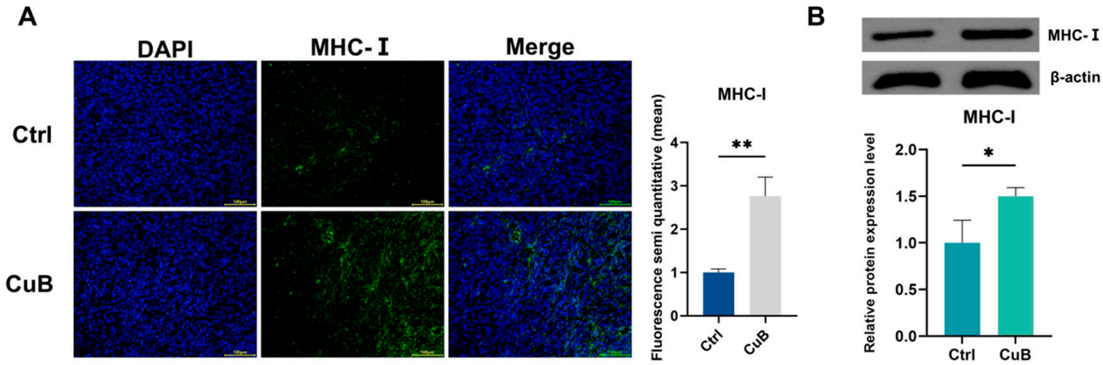

Fig. S4. The IF staining (A) and protein expression of MHC-I in tumor tissue (n=3). Bars, ± SE; Statistical significance was determined by an unpaired two-tailed Student's t-test; *p<0.05, **p<0.01.
